# Supplementary figures and images for: Testicular histone hyperacetylation in mice by valproic acid administration affects the next generation by changes in sperm DNA methylation
Source: PLoS One. 2023 Mar 9;18(3):e0282898. doi: 10.1371/journal.pone.0282898 (PMC9997898; doi:10.1371/journal.pone.0282898)

Day0

Day3

Day7

H3K9ac

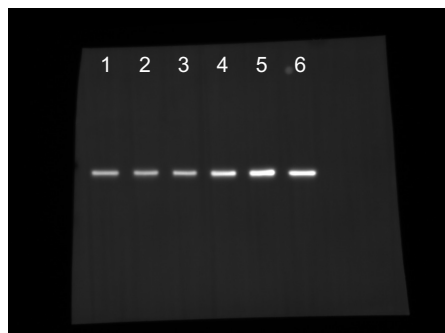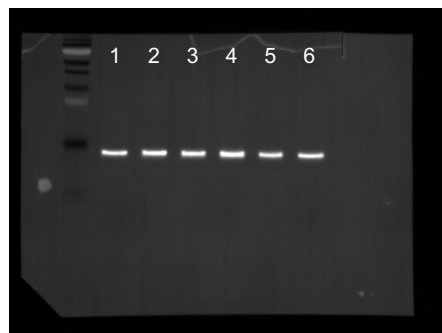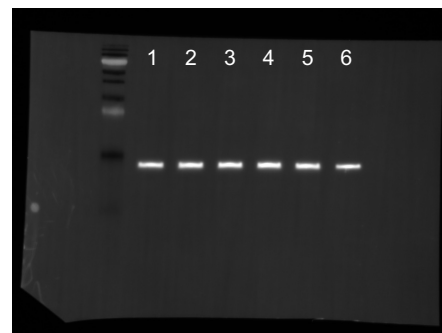

H3

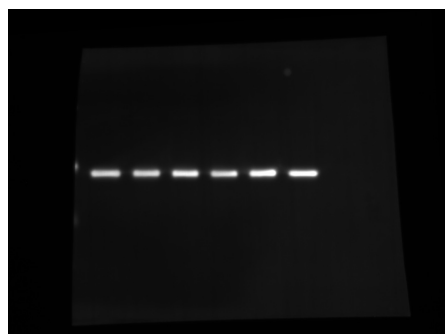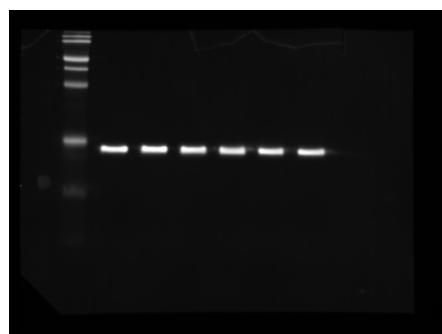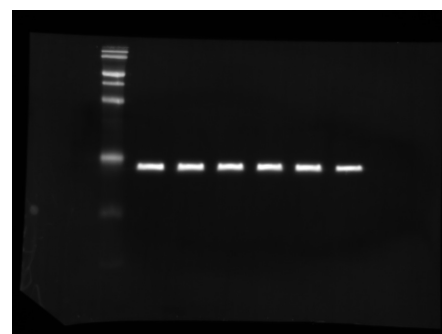

Merge

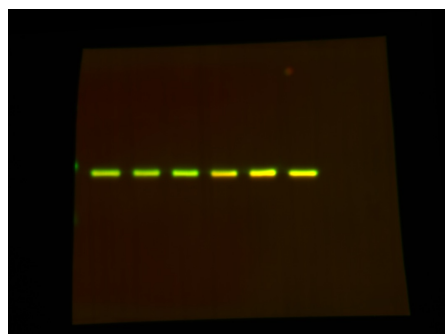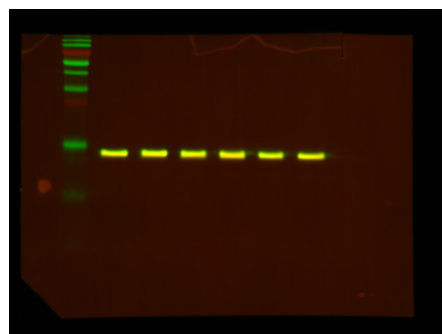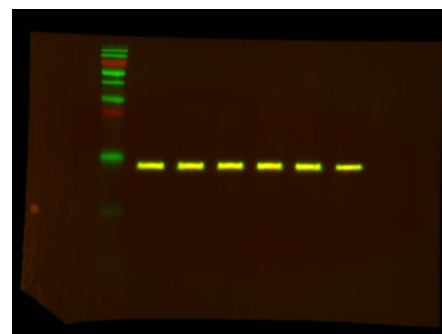

Supplement: S1 Raw image — The three lanes from the left (lane number 1–3) are the bands of the control group and the three lanes from the right (lane number 4–6) are the bands of the VPA-treated group. (PDF) [file pone.0282898.s001.pdf]

Day0

Day3

Day7

H3K27ac

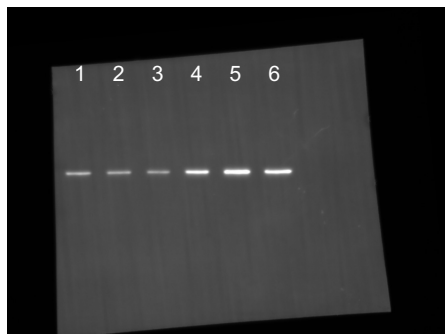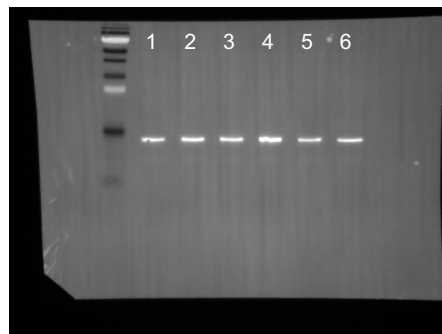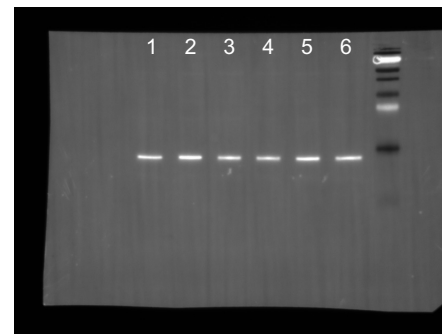

H3

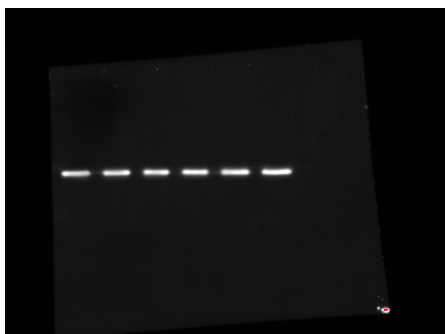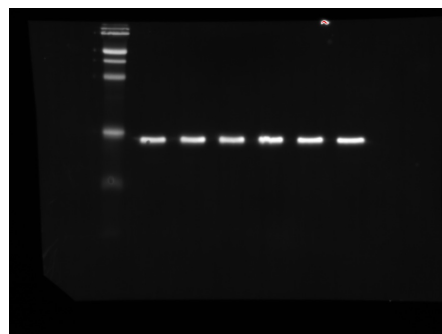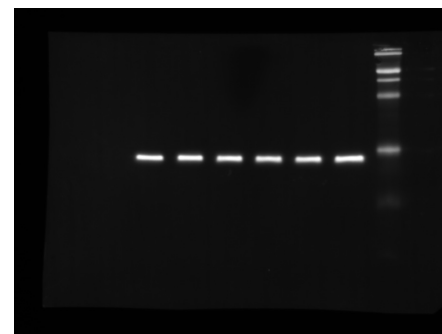

Merge

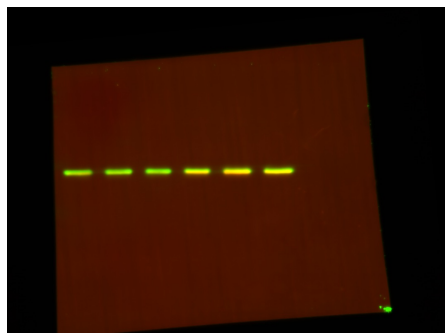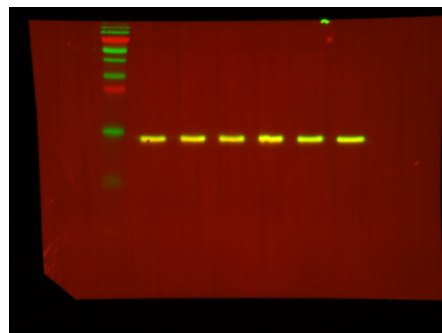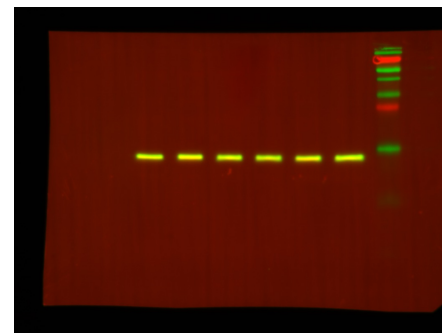

Supplement: S2 Raw image — The three lanes from the left (lane number 1–3) are the bands of the control group and the three lanes from the right (lane number 4–6) are the bands of the VPA-treated group. (PDF) [file pone.0282898.s002.pdf]
