# Supplementary material for: Testicular histone hyperacetylation in mice by valproic acid administration affects the next generation by changes in sperm DNA methylation
Source: PLoS One. 2023 Mar 9;18(3):e0282898. doi: 10.1371/journal.pone.0282898 (PMC9997898; doi:10.1371/journal.pone.0282898)
Supplement: Attachment — Submitted filename: Response to Reviewers.docx [file pone.0282898.s004.docx]

**Dear Editors and Reviewers**

Thank you very much for reviewing our manuscript and offering valuable advice.

We have addressed your comments with point-by-point responses, and revised the manuscript accordingly.

**Response to reviewer 1:**

We wish to express our appreciation to the Reviewer for his or her insightful comments, which have helped us significantly improve the paper.

**Comment:** *All comments raised has been satisfactorily addressed except the first on which the conclusion is based. The authors conclude that, "These findings suggest that VPA-induced histone hyperacetylation may have brain function-related effects on the next generation through changes in sperm DNA methylation. Demonstrating presence of acetylated histone H3 in the vicinity of the DMR based on available dataset does not suggest that histone hyperacetylation affects DNA methylation. In fact, recent reports suggests that "VPA as an HDAC inhibitor does not induce changes only in histone acetylation, but also changes in the state of DNA modification. It shows cross-reactivity between chromatin remodeling due to histone acetylation and DNA methylation" ( Cross-reactivity between histone demethylase inhibitor valproic acid and DNA methylation in glioblastoma cell lines Anna-Maria Barciszewska et al; Front Oncol. 2022 Nov 16;12:1033035. doi: 10.3389/fonc.2022.1033035.) The authors need to include this point in the manuscript.*

**Response:** We appreciate the helpful suggestion of the Reviewer. Although the report presented by the reviewer is the result of glioblastoma cells only, we also believe that it is a very important point. Within the manuscript, we also mention that the effect of VPA itself on DNA methylation should be considered (lines 446-448:” We believe that this phenomenon is due to the synergistic effects of chromatin relaxation, which promotes epigenetic changes, and the pharmacological effects of VPA itself, such as HDAC inhibition and involvement in GABA signaling.”). However, thanks to the reviewer's comments, we recognized the need for more detailed mention and have added the following text to the Discussion section:

“Since a recent study suggests that VPA affects DNA modification independently of histone acetylation, future studies should validate our results with HDAC inhibitors other than VPA, such as trichostatin A [44].” (page 14, lines 450-51.)

**Response to reviewer 2:**

We wish to express our strong appreciation to the reviewer for his or her insightful comments on our paper.

**Comment 1:** *Sperm DNA extracted for methylation analysis from VPA-treated mice was of which day after treatment- day 0, 3, 7?*

**Response:** We performed sperm DNA methylation analysis using mice two weeks after the last dose date. We apologize for our insufficient explanation and have added the following sentence to the relevant section:

“Two weeks after the final dose, 38 animals were sacrificed at 12 weeks of age and used for in vitro fertilization and sperm DNA methylation analysis.” (page 3, lines 90-92.)

**Comment 2**: *They have used morula stage for checking methylation level for reference to F1 generation. Why morula stage? DNA undergoes two time epigenetic reprogramming, first immediate fertilization and second at embryo7.25 stage in PGCs. So morula has just crossed first epigenetic reprogramming.*

**Response:** The reviewer correctly pointed out the important matter. If sperm DNA methylation is transmitted to the next generation, it should remain against epigenetic reprogramming immediately after fertilization. Therefore, we wanted to examine fertilized eggs immediately after fertilization, but we performed IVC because the number of cells (= amount of DNA) for analysis is very small. Meanwhile, as fertilized eggs are grown to blastocysts and beyond, the next generation's unique DNA methylation is established, making the interpretation of results at this stage more difficult. Therefore, we performed our analysis at the morula stage, a stage in which the number of cells is high and DNA methylation of parental origin appears to have been eliminated.

**Comment 3:** *Similarly, it goes for F1 generation sperm, that these sperm cannot be used to depict methylation level of F2 generation as it has to undergo embryonic epigenetic reprogramming.*

**Response:** As stated in the manuscript, it has been suggested that some regions escape epigenetic reprogramming and be transmitted to the next generation. The phenomenon of conservation of DNA methylation changes at some loci over multiple generations has been reported (Belleau P et al., Inferring and modeling inheritance of differentially methylated changes across multiple generations, Nucleic Acids Research. 2018. https://doi.org/10.1093/nar/gky362). It is also well known that epigenomic changes in sperm can cause phenotypic changes over multiple generations, and our results are important for investigating the mechanisms of such phenomena.

**Comment 4:** *In behavioural test (fear), conditioning for 1 day does not seem to be sufficient also why behavioural test has been done only in F1 generation and not in F0.*

**Response:** Our fear conditioning follows the general protocol and one day of conditioning is sufficient (Curzon P, Rustay NR, Browman KE. Cued and Contextual Fear Conditioning for Rodents. In: Buccafusco JJ, editor. Methods of Behavior Analysis in Neuroscience. 2nd edition. Boca Raton (FL): CRC Press/Taylor & Francis; 2009. Chapter 2. Available from: https://www.ncbi.nlm.nih.gov/books/NBK5223/).

VPA has been reported to affect memory performance through its pharmacological effects (Sintoni S et al., Chronic valproic acid administration impairs contextual memory and dysregulates hippocampal GSK-3β in rats. Pharmacology Biochemistry and Behavior. 2013 https://doi.org/10.1016/j.pbb.2013.02.013.). Therefore, we did not perform behavioral experiments on F0 mice, as this would only make interpretation of the experimental results more difficult.

Again, thank you for giving us the opportunity to strengthen our manuscript with your valuable comments and queries. We have worked hard to incorporate your feedback and hope that these revisions persuade you to accept our submission.
